# Epidemiological Characteristics and Spatiotemporal Analysis of Occupational Noise–Induced Deafness From 2006 to 2022 in Guangdong, China: Surveillance Study

**DOI:** 10.2196/57851

**Published:** 2024-11-29

**Authors:** Shanyu Zhou, Yongshun Huang, Lin Chen, Xianzhong Wen, Shu Wang, Lang Huang, Xudong Li

**Affiliations:** 1 Laboratory of Key Technology Research, Guangdong Province Hospital for Occupational Disease Prevention and Treatment, Guangzhou, China; 2 General Office, Guangdong Province Hospital for Occupational Disease Prevention and Treatment, 68 Haikang Street Xingang Road, Guangzhou, 510300, China, 86 20-89022988; 3 Occupational Health Evaluation and Monitoring Center, Guangzhou Twelfth People’s Hospital, Guangzhou, China; 4 Department of Technology Extension, Guangdong Province Hospital for Occupational Disease Prevention and Treatment, 68 Haikang Street Xingang RoadGuangzhou, 510300, China

**Keywords:** occupational noise-induced deafness, epidemiological characteristics, joinpoint regression, spatial autocorrelation, Guangdong, noise-induced, deafness, hearing loss, hearing impairment, occupational noise, noise, ONID, China, epidemiology, spatiotemporal analysis, comprehensive analysis, occupational diseases, policy formulation, health resource, surveillance, Moran's I, spatial, clustering, public health, teleaudiology, audiology

## Abstract

**Background:**

Occupational noise–induced deafness (ONID) has replaced occupational poisoning as the second most common occupational disease in China since 2015. However, there is a limited number of articles on epidemiological characteristics of legally diagnosed ONID.

**Objective:**

We conducted a comprehensive analysis of the epidemiological and spatiotemporal characteristics of ONID in Guangdong Province from 2006 to 2022, with the aim of providing a scientific foundation for policy formulation and health resource allocation.

**Methods:**

Surveillance data of ONID cases in Guangdong Province from 2006 to 2022 were obtained from the “Occupational Diseases and Health Hazard Factors Monitoring Information System.” Joinpoint regression analysis was applied to assess the long-term trends in cases of ONID from 2006 to 2022. Global spatial autocorrelation analysis was performed to measure the overall degree of similarity of the attribute values of spatially adjacent or neighboring regional units. The local indicators of spatial autocorrelation (LISA) plots were then used to identify the local clusters of ONID in Guangdong.

**Results:**

There were 3761 ONID cases in Guangdong Province from 2006 to 2022, showing a significantly increased trend in cases across the entire study period (average annual percentage change 21.9, 95% CI 18.7-35.1). The Moran’s I values for the period of 2006 to 2022 ranged from 0.202 to 0.649 (all *P*<.001), indicating a positive spatial correlation of ONID across regions each year in Guangdong Province. A total of 15 high-high clusters were notably concentrated in specific counties within the Pearl River Delta.

**Conclusions:**

Significant spatiotemporal patterns of ONID in Guangdong Province from 2006 to 2022 were identified, characterized by a dramatic increase followed by stabilization in case numbers. ONID predominantly occur in manufacturing industries, domestically funded enterprises, among males, individuals aged 40‐49 years, and those with 5+ years of occupational noise exposure. Spatial analysis demonstrated significant clustering in the Pearl River Delta region, with consistent positive spatial autocorrelation across years. These results could help prioritize the allocation of resources for targeted prevention and control measures for ONID.

## Introduction

Occupational noise is a common physical hazard that is considered loud or hazardous when it reaches 85 A-weighted Decibels (dBA) or higher in industrial working environments [1]. Prolonged exposure to excessive noise in the workplace could lead to occupational noise–induced deafness (ONID), a sensorineural hearing impairment that manifests as a high-frequency hearing loss during its early stages and gradually progresses to affect speech frequencies [2]. ONID, also known as occupational noise–induced hearing loss, is one of the most prevalent recognized occupational diseases in industrialized countries [3]. It represents a significant global health concern, affecting millions of workers worldwide. The Global Burden of Disease (GBD) study 2019 reported that approximately 1.57 billion individuals, or 1 in every 5 people, globally experienced hearing loss in 2019 [4]. World Report on Hearing released by the World Health Organization (WHO) estimated that by 2050, nearly 2.5 billion people will be living with some degree of hearing loss [5]. Notably, occupational noise exposure is responsible for 16% of disabling hearing loss cases worldwide [6]. In China, the situation is particularly concerning, with ONID becoming the second most common occupational disease since 2015 [7,8]. The legally reported cases of ONID were 11,811 from 2001 to 2019 [9], with an annual increase of 14.13%.

The burden of hearing loss owing to occupational noise is increasing and growing [10], with the years lived with disability of occupational noise-induced hearing loss increasing from 3.93 million in 1990 to 7.00 million in 2019 [4,11]. This trend is alarming, considering the widespread exposure to hazardous noise levels in workplaces. Recent estimates suggest that approximately 600 million workers globally are exposed to hazardous occupational noise levels [7]. This global issue is particularly pronounced in industrialized nations and regions, with an estimated 72 million workers exposed to harmful noise levels in the European Union [12], 22 million in the United States [1], and 80 million in China [9]. Notably, the WHO and International Labour Organization (ILO) joint estimates of the work-related burden of disease and injury indicate that the pooled prevalence of any high occupational noise exposure (≥85 dBA) in the general worker population is 0.17 [13]. This prevalence underscores the widespread nature of occupational noise exposure and highlights the potential scale of ONID as a public health challenge.

Previous research has mainly focused on the prevalence and global burden of occupational noise—induced hearing loss at global and national levels [7,11,14-16]. At the provincial level, studies have concentrated on the epidemiological distribution of legally reported ONID. However, there is a lack of research that simultaneously analyzes and uses spatiotemporal epidemiological methods to examine the epidemiological and spatiotemporal characteristics of ONID.

Guangdong Province, one of China’s most industrialized regions, was estimated to have 5.66 million noise-exposed workers in key industries, accounting for 17.36% of the country. Despite the substantial number of workers at high risk of ONID in Guangdong, the epidemiological and spatiotemporal characteristics of ONID are not clear, and the number of literature reviews on this topic is limited. Therefore, we conducted a comprehensive analysis of the epidemiological and spatiotemporal characteristics of ONID in Guangdong Province from 2006 to 2022. We innovatively applied spatiotemporal epidemiological methods, including joinpoint regression analysis and spatial autocorrelation analysis, to investigate ONID spatiotemporal patterns and identify high-risk ONID clusters. This study aims to provide a scientific basis for policy formulation and health resource allocation of ONID, and the implementation of Hearing Protection Actions in China and other low- and middle-income countries.

## Methods

### Study Area

**Figure 1.**
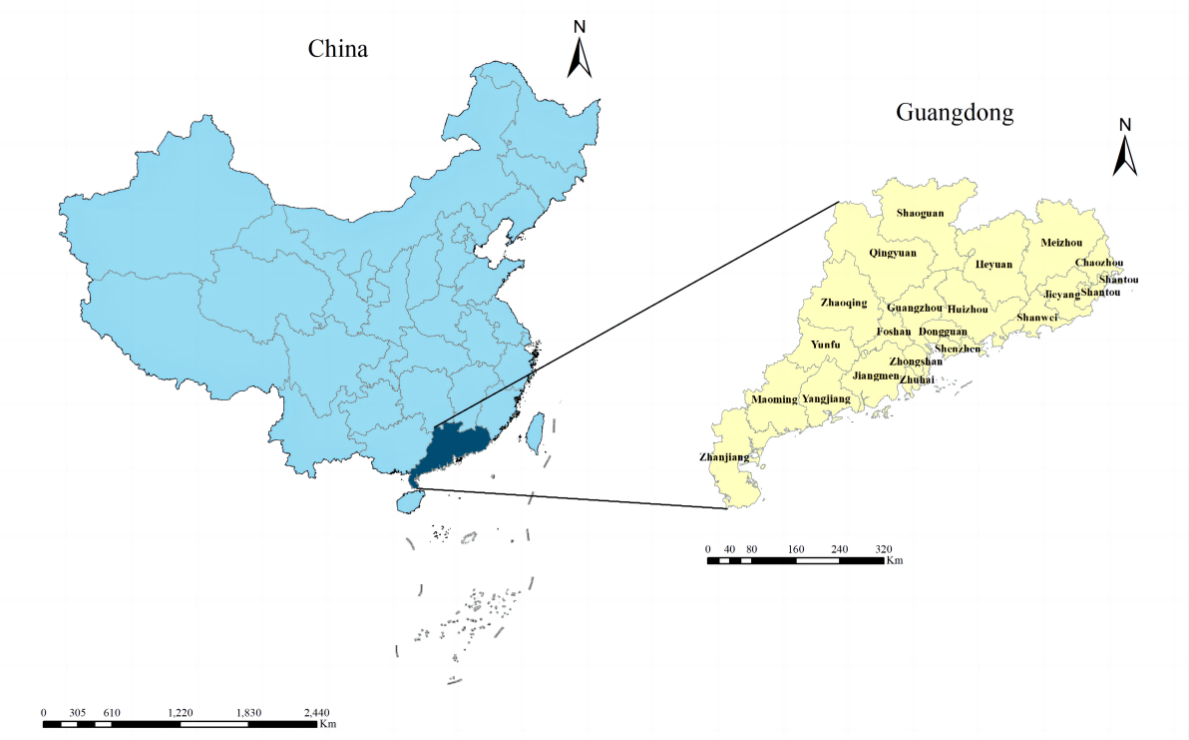
The geographical location of Guangdong Province in China.

Guangdong Province (109° 45' to 117° 20' E, 20° 09' to 25° 31' N) is located in the southern part of China and is a key component of the Guangdong-Hong Kong-Macau Greater Bay Area. Guangdong Province is divided into four regions: the Pearl River Delta, Eastern Wing, Western Wing, and mountainous areas. The Pearl River Delta includes Guangzhou, Shenzhen, Zhuhai, Foshan, Huizhou, Dongguan, Zhongshan, Jiangmen, and Zhaoqing. The non–Pearl River Delta regions consist of the Eastern Wing, Western Wing, and mountainous areas. Specifically, the Eastern Wing comprises Shantou, Shanwei, Chaozhou, and Jieyang; the Western Wing consists of Yangjiang, Zhanjiang, and Maoming; and the mountainous areas include Shaoguan, Heyuan, Meizhou, Qingyuan, and Yunfu. According to the Guangdong Statistical Yearbook 2022, Guangdong Province covers an area of 179,800 km^2^ with a resident population of 126.84 million and a population density of 706 people per km^2^ in 21 administrative cities and 122 counties [17]. All these areas were included in our study (Figure 1).

### Data Source

The surveillance data of ONID cases in Guangdong Province from January 1, 2006, to December 31, 2022, were procured from the “Occupational Diseases and Health Hazard Factors Monitoring Information System,” a subsystem of the “China Information System for Disease Prevention and Control.” All ONID cases were diagnosed by certified physicians of occupational disease diagnostic institutions or occupational disease identification institutions in accordance with the national standard *“*Diagnosis of occupational noise-induced deafness (GBZ 49)” and reported to the system within 15 days [2,18]. To ensure the integrity and accuracy of the data, all reported data in the system were reviewed at the county, city, and provincial levels [19].

The collected data consisted of three sections: (1) personal information, including sex, age, and duration of occupational noise exposure; (2) enterprise information, including name, address, scale, registration type, and industry classification; (3) diagnostic information, including date of diagnosis, date of reporting, and name of occupational disease diagnostic institutions or identification institutions. All case data in this study were anonymized. The digital maps of China and Guangdong Province were obtained from the National Catalog Service for Geographic Information [20].

### Statistical Analysis

#### Joinpoint Regression Analysis

Joinpoint regression analysis was applied to assess the long-term trends in cases of ONID in Guangdong from 2006 to 2022. Joinpoint regression models can divide the longitudinal variations into different segments by piecewise regression and identify the segment trends with statistical significance [21]. We calculated the annual percentage change (APC) for each segment, the average annual percentage change (AAPC), and its 95% CI for the global trend. The increasing (APC and AAPC>0) and decreasing trends (APC and AAPC<0) were identified by the slope of APC and AAPC and its significance (*P*<.05) and the stable trends referred to nonsignificant APC and AAPC (*P*≥.05) [22].

#### Spatial Autocorrelation Analysis

##### Global Spatial Autocorrelation

Global spatial autocorrelation analysis was performed to measure the overall degree of similarity of the attribute values of spatially adjacent or neighboring regional units [23]. Moran’s I was used as an indicator to determine the presence or absence of spatial autocorrelation of ONID in Guangdong Province. Moran’s I generally ranges from −1 to +1. When the Moran’s I value is greater than 0, it indicates positive spatial autocorrelation. When the Moran’s I value is less than 0, it indicates negative spatial autocorrelation. When the Moran’s I value is equal to 0, it indicates a random distribution and no spatial autocorrelation [24].

##### Local Spatial Autocorrelation

Local spatial autocorrelation reflects the degree of correlation between each local unit and its neighboring units and is applied to identify high- and low-value clustering of local spatial locations [23]. The local indicators of spatial autocorrelation (LISA) plots were then used to identify the local clusters of ONID in Guangdong Province. The LISA plot can reflect five spatial cluster patterns: (1) “High-High” indicates regions with high value surrounded by regions with high value, which are highly epidemical regions; (2) “High-Low” indicates regions with high value surrounded by regions with low value; (3) “Low-High indicates regions with low value surrounded by regions with high value; (4) “Low-Low” indicates regions with low value that are surrounded by regions with low value, which are lowly epidemical regions; and (5) “Not significant” indicates that there is no spatial autocorrelation [25,26].

### Data Analysis

Descriptive epidemiological methods were performed to investigate the characteristics of ONID, which included analyzing the distributions of cases by year, region, population demographics (such as sex, age, and duration of occupational noise exposure), and enterprise features (such as industry, enterprises scale, and registration types). Data were summarized using median and percentile (*P_25_*, *P_75_*) for nonnormally distributed continuous variables, and frequencies (n) with percentages (%) for categorical variables. Statistical analyses were performed using the R program (version 4.3.0; R Development Core Team), while the heat map was plotted using the ggplot2 R package (version 3.4.2). Joinpoint regression was performed using Joinpoint software (version 5.0.2; National Cancer Institute). A maximum of 5 joinpoints were initially set for the analysis to allow flexibility in determining the optimal number of joinpoints. The Grid search method was used for model fitting, and the Weight Bayesian Information Criterion was used for model selection. ArcGIS software (version 10.8; ESRI) was used for spatial autocorrelation analysis, mapping, and visualization analysis. The significance level for all statistical tests was set at a two-sided probability (*P*) of no more than .05.

### Ethical Considerations

This study was approved by the Ethics Committee of Guangdong Province Hospital for Occupational Disease Prevention and Treatment (approval number GDHOD MEC 2022057), with a waiver of informed consent granted for this retrospective analysis of existing surveillance records. All data were deidentified before analysis, with personal identifiers replaced by unique study codes. Investigators accessing the data were bound by strict security and confidentiality protocols. No compensation was provided as the study involved no direct participant contact.

## Results

### Epidemiological Characteristics of ONID

A total of 3761 ONID cases in Guangdong Province were reported from 2006 to 2022 via the Occupational Diseases and Health Hazard Factors Monitoring Information System, with an annual growth rate of 21.27%. While the number of cases fluctuated from 2006 to 2011, an overall upward trend was observed. Since 2011, the number of cases has gradually increased year by year. From 2015, it increased rapidly, peaking at 548 cases in 2019, followed by a sudden drop to 344 cases in 2020. The number of cases has remained relatively stable between 2020 and 2022. The proportion of ONID cases in Guangdong increased significantly from 5% of the national total in 2006 to 35.24% in 2019, with a notable acceleration observed from 2014 onwards (see Table S1 in Multimedia Appendix 1).

As shown in Table 1, of the total reported cases of ONID (N=3761), 3,338 (88.8%) were male and 423 (11.2%) were female. The median age at diagnosis of all reported cases was 44 (IQR 37‐49) years, with the age group of 40‐49 years accounting for the majority of cases (1670/3761, 44.4%). The median duration of occupational noise exposure was 9 (5-13) years. ONID was mainly distributed in the group with the duration of occupational noise exposure of 5-9 years, with 1399/3761 (37.2%) cases.

**Table 1.** Epidemiological characteristics and the average annual percentage change of occupational noise–induced deafness cases in Guangdong Province, 2006‐2022.

Year		Total	2006	2007	2008	2009	2010	2011	2012	2013	2014	2015	2016	2017	2018	2019	2020	2021	2022	AAPC^a^(95% CI)
Total, N (%)		3761(100)	16(100)	12(100)	35(100)	40(100)	24(100)	68(100)	110(100)	119(100)	182(100)	259(100)	347(100)	409(100)	544(100)	548(100)	344(100)	354(100)	350(100)	21.9(18.7 to 35.1)^b^
**Region, n (%)**																				
	Pearl River Delta	3581(95.2)	16(100)	12(100)	33(94.3)	38(95)	24(100)	67(98.5)	108(98.2)	117(98.3)	181(99.5)	251(96.9)	339(97.7)	394(96.3)	516(94.9)	520(94.9)	332(96.5)	328(92.7)	305(87.1)	21.2(16.3 to 25.2)^b^
	Non–Pearl River Delta	180(4.8)	0(0)	0(0)	2(5.7)	2(5)	0(0)	1(1.5)	2(1.8)	2(1.7)	1(0.5)	8(3.1)	8(2.3)	15(3.7)	28(5.1)	28(5.1)	12(3.5)	26(7.3)	45(12.9)	32.9(23.1 to 43.5)^b^
**Sex, n (%)**																				
	Male	3338(88.8)	16(100)	11(91.7)	31(88.6)	34(85)	22(91.7)	61(89.7)	99(90)	110(92.4)	157(86.3)	228(88)	310(89.3)	362(88.5)	485(89.2)	485(88.5)	309(89.8)	309(87.3)	309(88.3)	21.6(16.5 to 25.7)^b^
	Female	423(11.2)	0(0)	1(8.3)	4(11.4)	6(15)	2(8.3)	7(10.3)	11(10)	9(7.6)	25(13.7)	31(12)	37(10.7)	47(11.5)	59(10.8)	63(11.5)	35(10.2)	45(12.7)	41(11.7)	26.7(18.7 to 35.1)^b^
**Age (years), n (%)**																				
	<30	275(7.3)	5(31.3)	3(25)	8(22.9)	7(17.5)	0(0)	13(19.1)	22(20)	13(10.9)	30(16.5)	40(15.4)	27(7.8)	28(6.8)	24(4.4)	14(2.6)	12(3.5)	16(4.5)	13(3.7)	10.4(–8.3 to 32.9)
	30‐39	1058(28.1)	11(68.8)	7(58.3)	19(54.3)	16(40)	17(70.8)	28(41.2)	44(40)	56(47.1)	68(37.4)	78(30.1)	107(30.8)	124(30.3)	142(26.1)	122(22.3)	81(23.5)	79(22.3)	59(16.9)	12.5(8.8 to 15.7)^b^
	40‐49	1670(44.4)	0(0)	1(8.3)	7(20)	15(37.5)	5(20.8)	22(32.4)	35(31.8)	38(31.9)	64(35.2)	108(41.7)	161(46.4)	196(47.9)	275(50.6)	281(51.3)	163(47.4)	152(42.9)	147(42)	43.5(33.9 to 53.7)^b^
	≥50	758(20.2)	0(0)	1(8.3)	1(2.9)	2(5)	2(8.3)	5(7.4)	9(8.2)	12(10.1)	20(11)	33(12.7)	52(15)	61(14.9)	103(18.9)	131(23.9)	88(25.6)	107(30.2)	131(37.4)	40.7(35.3 to 45.1)^b^
**Duration of occupational noise exposure (years), n (%)**																				
	<5	672(17.9)	4(25)	1(8.3)	9(25.7)	9(22.5)	2(8.3)	21(30.9)	32(29.1)	30(25.2)	46(25.3)	57(22)	76(21.9)	97(23.7)	105(19.3)	69(12.6)	34(9.9)	38(10.7)	42(12)	18.2(6.5 to 30.5)^c^
	5-9	1399(37.2)	4(25)	5(41.7)	12(34.3)	14(35)	10(41.7)	18(26.5)	38(34.5)	37(31.1)	74(40.7)	94(36.3)	136(39.2)	140(34.2)	213(39.2)	208(38)	134(39)	136(38.4)	126(36)	23(17.7 to 27.2)^b^
	10-14	892(23.7)	8(50)	4(33.3)	11(31.4)	13(32.5)	8(33.3)	19(27.9)	23(20.9)	25(21)	32(17.6)	51(19.7)	77(22.2)	102(24.9)	117(21.5)	142(25.9)	82(23.8)	92(26)	86(24.6)	18.6(13.5 to 23.6)^b^
	≥15	798(21.2)	0(0)	2(16.7)	3(8.6)	4(10)	4(16.7)	10(14.7)	17(15.5)	27(22.7)	30(16.5)	57(22)	58(16.7)	70(17.1)	109(20)	129(23.5)	94(27.3)	88(24.9)	96(27.4)	35.7(30.5 to 41.1)^b^
**Industries, n (%)**																				
	Manufacturing Industry	3513(93.4)	15(93.8)	12(100)	35(100)	39(97.5)	24(100)	63(92.6)	108(98.2)	115(96.6)	178(97.8)	222(85.7)	320(92.2)	377(92.2)	498(91.5)	517(94.3)	326(94.8)	334(94.4)	330(94.3)	21.6(16.7 to 25.6)^b^
	Nonmanufacturing Industry	248(6.6)	1(6.3)	0(0)	0(0)	1(2.5)	0(0)	5(7.4)	2(1.8)	4(3.4)	4(2.2)	37(14.3)	27(7.8)	32(7.8)	46(8.5)	31(5.7)	18(5.2)	20(5.6)	20(5.7)	27.5(14.7 to 43.5)^b^
**Enterprises scales, n (%)**																				
	Large	1044(27.8)	7(43.8)	5(41.7)	12(34.3)	16(40)	10(41.7)	17(25)	34(30.9)	40(33.6)	69(37.9)	107(41.3)	105(30.3)	105(25.7)	157(28.9)	116(21.2)	106(30.8)	82(23.2)	56(16)	16.1^b^(10.8 to 20.6)
	Medium	1361(36.2)	3(18.8)	3(25)	16(45.7)	19(47.5)	10(41.7)	31(45.6)	46(41.8)	53(44.5)	74(40.7)	82(31.7)	126(36.3)	161(39.4)	179(32.9)	194(35.4)	109(31.7)	119(33.6)	136(38.9)	23.4(16.0-30.4)^b^
	Small	1161(30.9)	0(0)	1(8.3)	2(5.7)	4(10)	4(16.7)	18(26.5)	30(27.3)	23(19.3)	35(19.2)	62(23.9)	105(30.3)	116(28.4)	177(32.5)	210(38.3)	104(30.2)	127(35.9)	143(40.9)	40.8(35.2 to 46.7)^b^
	Minor and unknown	195(5.2)	6(37.5)	3(25)	5(14.3)	1(2.5)	0(0)	2(2.9)	0(0)	3(2.5)	4(2.1)	8(3.1)	11(3.2)	27(6.6)	31(5.7)	28(5.1)	25(7.2)	26(7.4)	15(4.3)	5.6(–3.4 to 14.1)
**Registration types, n (%)**																				
	Domestic-funded	1704(45.3)	10(62.5)	8(66.7)	9(25.7)	10(25)	10(41.7)	21(30.9)	34(30.9)	34(28.6)	50(27.5)	113(43.6)	145(41.8)	208(50.9)	252(46.3)	281(51.3)	130(37.8)	175(49.4)	214(61.1)	21(14.6 to 27.3)^b^
	Hong Kong, Macao, and Taiwan-funded	1113(29.6)	2(12.5)	2(16.7)	15(42.9)	14(35)	7(29.2)	23(33.8)	27(24.5)	32(26.9)	66(36.3)	64(24.7)	113(32.6)	107(26.2)	151(27.8)	160(29.2)	144(41.9)	109(30.8)	77(22)	23.3(13.5 to 32.7)^b^
	Foreign-funded	944(25.1)	4(25)	2(16.7)	11(31.4)	16(40)	7(29.2)	24(35.3)	49(44.5)	53(44.5)	66(36.3)	82(31.7)	89(25.6)	94(23)	141(25.9)	107(19.5)	70(20.3)	70(19.8)	59(16.9)	21.6(13.2 to 30.5)^b^

a AAPC: average annual percentage change.

b *P*<.001.

c *P*=.005.

Regarding the industries, the cases were mainly distributed in manufacturing (3513/3761 cases, 93.4%). Among the major categories of manufacturing industries, the top five with the highest number of reported cases of ONID are (1) the metal products industry (612/3513, 17.4% cases); (2) the computer, communication, and other electronic equipment manufacturing industry (362/3513, 10.3% cases); (3) the electrical machinery and equipment manufacturing industry (239/3513, 6.8% cases); (4) the general equipment manufacturing industry (224/3513, 6.4% cases); and (5) the nonmetallic mineral products industry (207/3513, 5.9% cases; Table S2 in Multimedia Appendix 1). In addition, an analysis of the annual distribution of ONID cases across manufacturing industries from 2006 to 2022 showed changes over time (Table S3 in Multimedia Appendix 1). The metal products industry and the computer, communication, and other electronic equipment manufacturing industries have become consistently prominent in recent years. In contrast, some industries that frequently appeared in the top 5 during earlier years, such as transportation equipment manufacturing, were less represented in later years.

In terms of enterprises scale, there were 1361 (36.2%) cases in medium enterprises, 1161 (30.9%) cases in small enterprises, 1044 (27.8%) cases in large enterprises, and 195 (5.2%) in micro- and unknown enterprises. When enterprises were categorized by registration type, the highest number of reported cases was in domestic-funded enterprises (1704/3761, 45.3%), followed by Hongkong, Macau, and Taiwan-funded enterprises (1113/3761, 29.6%) and foreign-funded enterprises (944/3761, 25.1%).

**Figure 2.**
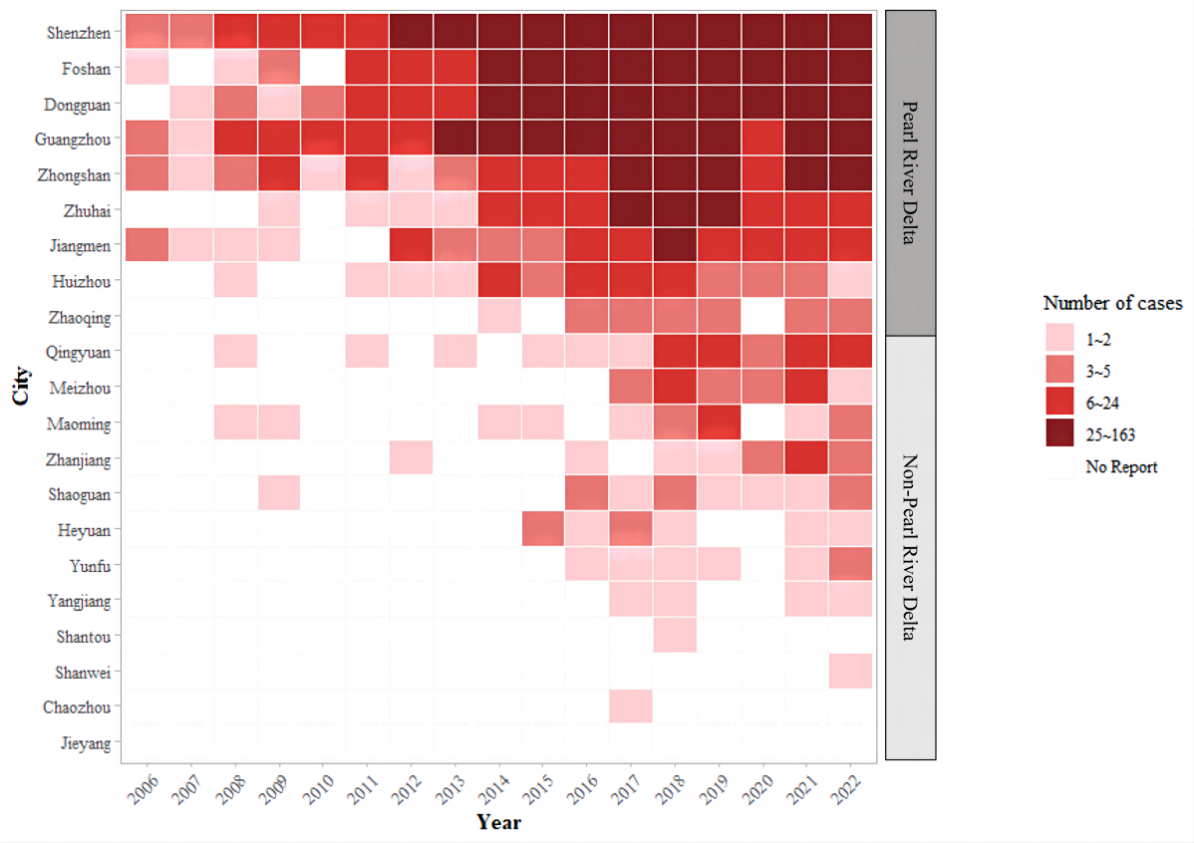
Heat map of yearly occupational noise–induced deafness for each city by regions during 2006‐2022.

Figure 2 displays the heat map of yearly ONID for each city from 2006 to 2022. Almost all cases (3580/3761, 95.2%) occurred in the Pearl River Delta, particularly in Shenzhen, Guangzhou, Foshan, Dongguan, and Zhongshan, accounting for 84.1% (3167/3761). Since 2015, the epidemic has slowly expanded to the non–Pearl River Delta region. Although sporadic cases have been reported in the non–Pearl River Delta region, the number of cases has increased since 2015. The number of affected cities has increased from 5 in 2006 to 19 in 2022. By 2022, a total of 20 cities (3580/3761, 95.2%) and 80 counties (2469/3761, 65.6%) in Guangdong Province had reported cases of ONID.

### Temporal Trends of ONID by Subgroups

Table 1 displays the AAPC and its 95% CI of ONID in Guangdong from 2006 to 2022, stratified by various subgroups such as demographic factors (sex, age, and duration of occupational noise exposure), geographic factors (regions), and enterprise factors (industries, registration types, and enterprises scales). Overall, ONID showed a significantly increased trend in cases across the entire study period (AAPC 21.9, 95% CI 18.7-35.1). When stratified by regions, AAPC was 21.2 (95% CI 16.3-25.2) in the Pearl River Delta and 32.9 (95% CI 23.1-43.5) in the non–Pearl River Delta.

**Figure 3.**
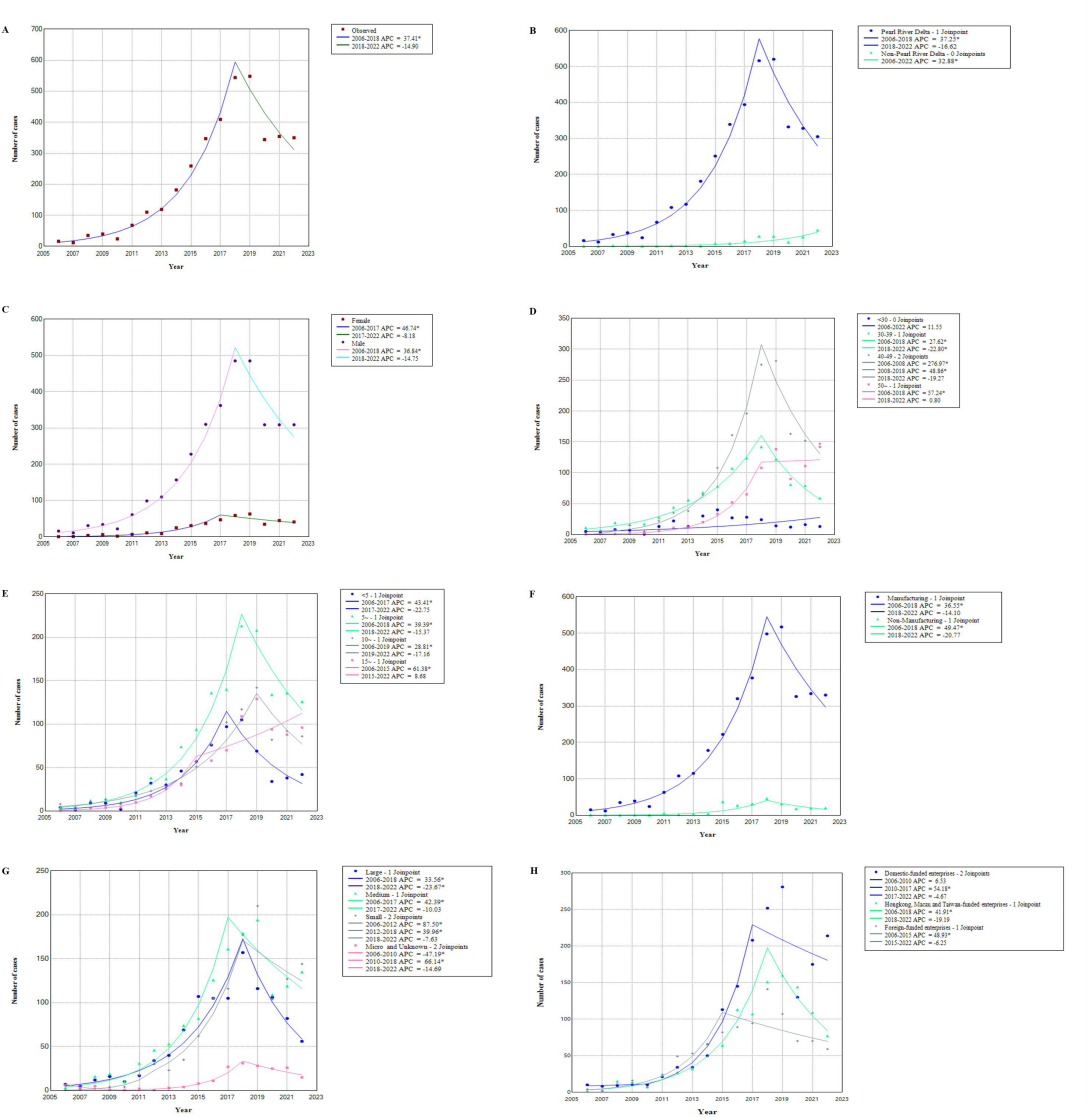
Trends of occupational noise–induced deafness in Guangdong during 2006‐2022. (**A**) Overall cases. (**B**) Stratified by regions. (**C**) Stratified by sex. (**D**) Stratified by age. (**E**) Stratified by duration of occupational noise exposure. (**F**) Stratified by industries. (**G**) Stratified by enterprise scales. (**H**) Stratified by registration types. Asterisk (*) indicates that annual percentage change is significantly different from zero at the α=.05 level. APC: annual percentage change.

As shown in Figure 3, ONID cases increased from 2006 to 2018 with an associated APC of 37.41 (95% CI 31.8-44.3), followed by a period without significant change between 2019 and 2022. Further analyses in cases of ONID in subgroups exhibited similar patterns.

### Spatial Autocorrelation Analysis

The global spatial autocorrelation analyses of occupational noise–induced hearing loss in Guangdong Province during 2006‐2022 are shown in Table 2. The Moran’s I values for the period of 2006 to 2022 ranged from 0.202 to 0.649 (all *P*<.001), with the highest value observed in 2013 at 0.649 and the lowest value in 2006 at 0.202, indicating that ONID in Guangdong province was positively spatially correlated across regions in each year.

**Figure 4.**
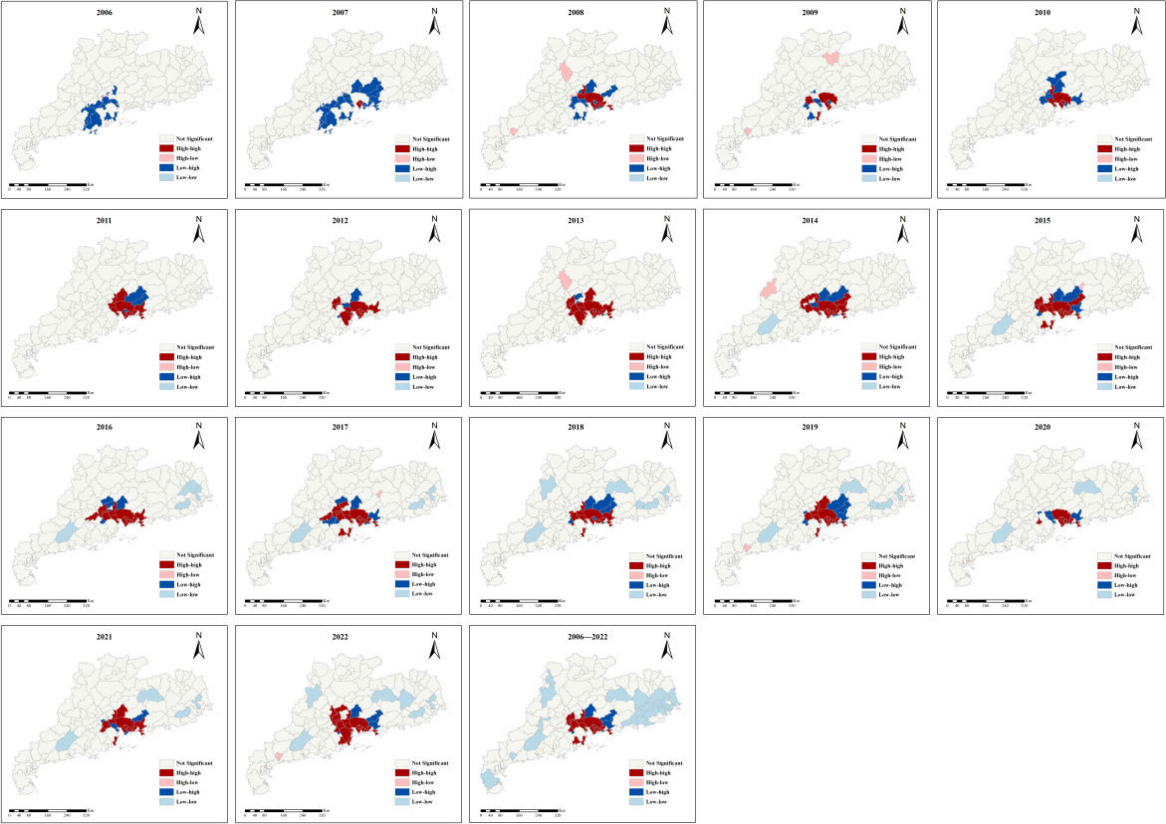
Local indicators of spatial autocorrelation maps of occupational noise–induced deafness cases in Guangdong Province at county level, 2006‐2022.

Further results of the local autocorrelation analysis are presented in Figure 4. According to the annual LISA cluster maps, the number of counties with high-high and low-low clustering areas gradually increased from 2006 to 2022, while the number of low-high clustering decreased. As for the total noise-induced noise deafness cases, the spatial clustering characteristics of noise-induced noise deafness in Guangdong at the county level are mainly characterized by high-high, low-low, and low-high clustering. There are 15 high-high clustering, relatively concentrated in some counties of the Pearl River Delta, with 5 in Shenzhen, 3 in Guangzhou, 3 in Foshan, 2 in Zhuhai, and 1 in Jiangmen and Dongguan city. In total, 5 low-high clustering were surrounded by high-high clustering. A total of 22 low-low clustering areas were mainly concentrated in most counties of the Eastern Wing, as well as scattered in some counties of the Western Wing and mountainous areas.

**Table 2.** Global spatial autocorrelation analysis of occupational noise–induced deafness in Guangdong Province.

Year	Moran’s I	*z* score	*P* value
2006	0.202	3.388	<.001
2007	0.260	4.282	<.001
2008	0.352	5.660	<.001
2009	0.207	3.810	<.001
2010	0.378	6.265	<.001
2011	0.285	5.371	<.001
2012	0.454	7.542	<.001
2013	0.649	10.250	<.001
2014	0.520	8.395	<.001
2015	0.540	8.902	<.001
2016	0.572	9.068	<.001
2017	0.533	8.489	<.001
2018	0.303	5.488	<.001
2019	0.329	5.641	<.001
2020	0.548	9.083	<.001
2021	0.512	8.323	<.001
2022	0.421	6.873	<.001
Total	0.540	8.850	<.001

## Discussion

### Principal Findings

Based on the long-term surveillance data of ONID in Guangdong Province, we comprehensively overviewed the epidemiological characteristics of the disease in Guangdong from 2006 to 2022. Then the spatial autocorrelation analysis methods were used to explore the spatiotemporal clusters of ONID, which provided health policy makers with reference data to develop measures for preventing and controlling ONID.

ONID was listed as a reported occupational disease by the National Occupational Diseases and Hazards Monitoring Information System developed in 2006 [27]. From 2006 to 2022, a total of 3761 ONID in Guangdong Province were reported, with an annual growth rate of 21.27%. Although Guangdong Province leads the nation in cases of ONID, there is a significant gap between those diagnosed and the actual cases. A meta-analysis involving a total of 71,865 workers from transportation, mining, and typical manufacturing industries revealed that the general prevalence of occupational noise–induced hearing loss in China was 21.3%, of which noise-induced deafness accounted for 5.8% [7]. Our previous study estimated that approximately 12 million workers in Guangdong are exposed to hazardous noise at their workplaces. Therefore, the diagnosed and reported ONID might be just the tip of the iceberg.

Due to the revision of “Diagnosis of occupational noise-induced deafness (GBZ 49)” in 2014, more mild cases could be identified. The hearing threshold of 4000 Hz with a weight of 0.1 was included as the diagnostic hearing threshold and a new method was used to correct for age and gender [2,28]. Therefore, the reported cases of ONID in Guangdong Province have increased dramatically since 2015. The sudden decline in reported cases during 2019‐2022 might be attributed to the COVID-19 pandemic, which led to the closure of some businesses and fewer workers requiring an occupational disease diagnosis. There were also labor shortages as occupational health professionals were deployed to support the fight against COVID-19. These factors contributed to a decrease in diagnosed cases during the COVID-19 pandemic.

Previous studies have shown that the prevalence of occupational noise–induced hearing loss is generally higher in the less developed regions of the world [29]. However, ONID was more concentrated in the Pearl River Delta region, which is the more developed region of Guangdong. The Pearl River Delta region, as a globally significant hub of advanced manufacturing, exhibits a high spatial agglomeration of industrial enterprises [30]. The high number of cases of ONID in the Pearl River Delta may be attributed not only to the concentrated and severe nature of noise hazards in these areas but also to the high level of attention given to occupational disease prevention and control, leading to a higher rate of occupational health examinations for workers exposed to noise, thereby facilitating the early detection of ONID. In addition, more than 90% of the cases occurred in the manufacturing industries. This distribution differs from that reported in Henan Province, where mining and manufacturing industries accounted for 50% and 43.21% of cases, respectively [31]. Guangdong is known as a province with a strong focus on the manufacturing industries. According to the “Guangdong Statistical Yearbook 2023,” the number of manufacturing enterprises above the designated size in Guangdong Province is 70,725, ranking first in the country, with 85.27% of them located in the Pearl River Delta region [32,33]. Occupational noise exposure is relatively severe in manufacturing industries. The 2010 National Health Interview Survey (NHIS) data showed that 46.49% of workers in the manufacturing industries are exposed to occupational noise, and 18.32% report having hearing difficulty [34]. Therefore, the Pearl River Delta region and the manufacturing industries should be regarded as the key regions and industries for the prevention and control of ONID in Guangdong Province.

In terms of gender, a higher number of cases was observed in males, which is consistent with previous studies [7,35]. The gender discrepancy could be attributed to hormone-driven physiological differences in auditory sensitivity [14]. It could also be related to occupational differences between males and females. Male workers are more likely to be exposed to a noisy working environment [36]. For age distribution, most cases of ONID occurred in the age group 40‐49 years, with a median age of 44 (IQR 37-49) years. This corresponds to the peak labor force participation age [37] and is consistent with findings from other provinces: 45 years in Zhejiang [38], 46 years in Sichuan [39], and 47 years in Henan [31], Jiangsu [40], and Chongqing [41]. However, a notable difference was observed in Tianjin, where the average age of onset was reported to be significantly higher at 53 years. ONID can result from the cumulative effects of prolonged occupational noise exposure. In our study, the number of cases of ONID increased rapidly within 5-9 years of exposure and then reached a plateau after 10 years. The median duration of occupational noise exposure before ONID onset in Guangdong was found to be 9 years. This duration is comparable to those reported in Zhejiang (9 y) [38], Chongqing (10 y) [41], and Sichuan (11 y) [39], but notably shorter than the exposure periods observed in Henan (16 y) [31] and Tianjin (25 y) [42].

Joinpoint regression analysis of ONID revealed 2 distinct periods. ONID cases increased significantly from 2006 to 2018 with an associated APC of 37.41 (95% CI 31.8-44.3), followed by a period without significant change between 2019 and 2022. The year 2018 emerged as a significant inflection point in this trend. Before 2018, ONID cases showed a marked upward trend, while after 2018, particularly from 2020 onward, the number of cases stabilized and subsequently declined. This change can be attributed to several factors. In late 2017, the National Health and Family Planning Commission issued an official reply clarifying that the 3-year continuous work tenure required for ONID diagnosis should be calculated based on calendar days, already accounting for overtime. This clarification standardized the diagnostic criteria and potentially reduced the number of cases that would have only met the 3-year exposure threshold if overtime hours were included in the calculation. Although the number of cases in 2018 (554 cases) and 2019 (560 cases) did not show an immediate significant decrease, this interpretation may have influenced the long-term trend of ONID cases, leading to the inflection point observed in the joinpoint analysis. Furthermore, economic shifts and business closures in recent years have significantly contributed to the decline in ONID cases. The closure of many businesses has led to a decrease in the number of workers exposed to occupational noise, consequently reducing the number of potential ONID cases detected. The combined effect of these economic changes and the 2017 official interpretation of diagnostic criteria provides a plausible explanation for the stabilization and subsequent decline in ONID cases observed from 2020 onward.

The consistent positive global Moran’s I values (0.202‐0.649, *P*<.001) from 2006 to 2022 reveal significant spatial clustering of ONID cases across Guangdong Province, indicating that nearby areas tend to have similar ONID rates and suggesting that ONID risk factors are geographically influenced rather than randomly distributed. Annual LISA cluster maps suggested that the number of counties with high-high and low-low clustering areas gradually increased from 2006 to 2022, while the number of low-high clustering decreased. It indicated a notable shift in the spatial distribution pattern of ONID cases over the years. LISA maps identified 15 high-risk areas, which were primarily concentrated in developed cities including Shenzhen, Guangzhou, Foshan, Zhuhai, Dongguan, and Jiangmen. This suggests that these areas serve as hot spots for ONID, which should be prioritized for interventions, including enhanced workplace monitoring and adequate public hearing conservation resources [10]. The observed spatial clustering of ONID cases is closely associated with the concentration of high-noise industries in these areas. Metal products manufacturing, computer, communication and other electronic equipment manufacturing, and shipbuilding are characteristic industries in these regions. These industries are known for their high-intensity noise environments, which may contribute to the relative clustering of ONID cases. Moreover, the identified high-risk areas correspond to cities ranking highest in gross domestic product within Guangdong Province [32], suggesting that more economically developed areas have higher reported ONID cases. This could be due to better health care access, more advanced diagnostic capabilities, and stricter enforcement of occupational health regulations in these areas. It is worth noting, however, that the higher number of reported cases in these regions may reflect improved detection and reporting mechanisms rather than necessarily indicating a higher incidence rate.

A total of 5 low-high clusters were identified surrounded by high-high clustering, suggesting potential disparities in occupational health practices between adjacent regions or a gradual diffusion of risk factors from high-risk areas. Conversely, 22 low-low clusters were predominantly observed in the Eastern and Western Wings and mountainous areas, likely reflecting regional variations in industrial composition or occupational risk profiles. However, it is imperative to verify that these patterns are not artifacts of underreporting or less rigorous occupational health surveillance in these regions.

This study provides critical insights for health decision makers in Guangdong Province by highlighting the necessity for geographically tailored occupational health strategies to address ONID. By identifying high-risk areas, policy makers can prioritize resources and interventions effectively, such as using noise reduction strategies, enhancing health promotion for workers, implementing comprehensive screening programs, and improving accessibility to hearing protection devices in identified hotspots [4,9]. These targeted efforts are crucial, as ONID is a condition that is permanent yet entirely preventable with current hearing loss prevention strategies and technologies [43]. Our findings on the spatial distribution of ONID can also inform targeted public health campaigns and occupational health policies, leading to improved early detection and prevention strategies. Moreover, the observed temporal trends offer valuable guidance for long-term planning and evaluation of occupational health interventions. By addressing these spatial and temporal patterns, health authorities can work toward more equitable and effective occupational health protection across Guangdong Province, ultimately reducing the burden of ONID on the workforce and the associated economic costs.

This study represents the first endeavor to monitor ONID epidemic data in Guangdong Province by spatial autocorrelation analysis, providing a comprehensive exploration of its epidemic and spatial distribution characteristics. However, 3 limitations should be acknowledged. First, our study is based on data from the Occupational Diseases and Health Hazard Factors Monitoring Information System. Workers’ potential reluctance to initiate the occupational disease diagnostic procedure due to personal considerations might lead to underreporting of ONID cases, potentially affecting the comprehensiveness of our dataset. In addition, due to the absence of data on the number of workers in Guangdong who are exposed to noise, the incidence rate of ONID cannot be calculated. Nevertheless, Guangdong Province has initiated the “Occupational Disease Hazard Project Declaration Management Action” since 2023 to comprehensively ascertain the number of workers exposed to occupational hazards. Furthermore, our study only provided a description of epidemiology characteristics and spatial autocorrelation of ONID. Future studies should aim to collect essential influencing factors, such as the intensity of occupational noise exposure, socioeconomic factors, and detailed patient onset information, for inclusion. These additional data could offer further insights into the causal relationship of ONID onset.

### Conclusion

In conclusion, ONID in Guangdong Province has experienced a dramatic increase followed by a stabilization from 2006 to 2022, currently ranking at the top nationwide in terms of the number of cases. Joinpoint regression analysis revealed a significant upward trend in the early years, followed by a period of stabilization in recent years. ONID cases predominantly occurs in the manufacturing industries, within domestically funded enterprises, and are most prevalent among males, aged 40‐49 years, and those with 5-9 years of occupational noise exposure. Notably, the distribution of ONID was spatially clustered in the Pearl River Delta of Guangdong Province. Global spatial autocorrelation analysis consistently showed positive spatial correlation across regions each year, while local spatial autocorrelation analysis identified several high-risk clusters primarily concentrated in specific counties within the Pearl River Delta, including cities such as Shenzhen, Guangzhou, Foshan, Zhuhai, Jiangmen, and Dongguan. The findings from our data-driven study could be instrumental in determining the priorities for the precise and effective allocation of resources to prevent and control ONID.

## Supplementary material

10.2196/57851 Multimedia Appendix 1 Additional tables.

## Abbreviations

AAPC: average annual percentage change
APC: annual percentage change
dBA: A-weighted Decibels
dBA: decibels
GBD: Global Burden of Disease
ILO: International Labour Organization
LISA: local indicators of spatial autocorrelation
NHIS: National Health Interview Survey
ONID: occupational noise–induced deafness
WHO: World Health Organization
