# Supplementary material for: Epidemiological Characteristics and Spatiotemporal Analysis of Occupational Noise–Induced Deafness From 2006 to 2022 in Guangdong, China: Surveillance Study
Source: JMIR Public Health Surveill. 2024 Nov 29;10:e57851. doi: 10.2196/57851 (PMC11623259; doi:10.2196/57851)
Supplement: Multimedia Appendix 1 [file publichealth-v10-e57851-s001.docx]

**Table S1 Annual distribution of occupational noise-induced deafness (ONID) cases in Guangdong Province and China, 2006-2019**

| Year | Guangdong | Nation^a^ | Proportion (%) |
| --- | --- | --- | --- |
| 2006 | 16 | 320 | 5.00 |
| 2007 | 12 | 269 | 4.46 |
| 2008 | 35 | 223 | 15.70 |
| 2009 | 40 | 348 | 11.49 |
| 2010 | 24 | 333 | 7.21 |
| 2011 | 68 | 492 | 13.82 |
| 2012 | 110 | 597 | 18.43 |
| 2013 | 119 | 681 | 17.47 |
| 2014 | 182 | 825 | 22.06 |
| 2015 | 259 | 1052 | 24.62 |
| 2016 | 347 | 1220 | 28.44 |
| 2017 | 409 | 1536 | 26.63 |
| 2018 | 544 | 1464 | 37.16 |
| 2019 | 548 | 1555 | 35.24 |

^a^: National data sourced from: Sun X. Occupational Noise Exposure and Worker's Health in China. CCDCW China CDC Weekly. 2021 Apr 30;3(18):375–377. doi: 10.46234/ccdcw2021.102

**Table S2 The manufacturing industry distribution of ONID in Guangdong province from 2006 to 2022**

| **The Classification of Manufacturing Industry** | ***n*** | **%** |
| --- | --- | --- |
| Metal Products Industry | 612 | 17.42 |
| Computer, Communication, and Other Electronic Equipment Manufacturing Industry | 362 | 10.30 |
| Electrical Machinery and Equipment Manufacturing Industry | 239 | 6.80 |
| General Equipment Manufacturing Industry | 224 | 6.38 |
| Non-metallic Mineral Products Industry | 207 | 5.89 |
| Rubber and Plastic Products Industry | 203 | 5.78 |
| Special Equipment Manufacturing Industry | 203 | 5.78 |
| Railway, Shipbuilding, Aerospace, and Other Transportation Equipment Manufacturing Industry | 157 | 4.47 |
| Cultural, Educational, Arts, Sports, and Entertainment Products Manufacturing Industry | 155 | 4.41 |
| Printing and Reproduction of Recorded Media Industry | 127 | 3.62 |
| Other Manufacturing Industries | 117 | 3.33 |
| Automobile Manufacturing Industry | 116 | 3.30 |
| Furniture Manufacturing Industry | 108 | 3.07 |
| Chemical Raw Materials and Chemical Products Manufacturing Industry | 90 | 2.56 |
| Textile Industry | 81 | 2.31 |
| Leather, Fur, Feather and Related Products, and Footwear Industry | 78 | 2.22 |
| Non-ferrous Metal Smelting and Rolling Industry | 77 | 2.19 |
| Paper and Paper Products Industry | 71 | 2.02 |
| Food Manufacturing Industry | 55 | 1.57 |
| Instrumentation Manufacturing Industry | 47 | 1.34 |
| Petroleum, Coal, and Other Fuel Processing Industry | 32 | 0.91 |
| Ferrous Metal Smelting and Rolling Industry | 30 | 0.85 |
| Alcohol, Beverage, and Refined Tea Manufacturing Industry | 25 | 0.71 |
| Pharmaceutical Manufacturing Industry | 21 | 0.60 |
| Wood Processing and Wood, Bamboo, Rattan, Palm, and Straw Products Industry | 20 | 0.57 |
| Agricultural and Sideline Food Processing Industry | 19 | 0.54 |
| Repair of Metal Products, Machinery, and Equipment Industry | 15 | 0.43 |
| Textile, Apparel, and Accessories Industry | 10 | 0.28 |
| Chemical Fiber Manufacturing Industry | 8 | 0.23 |
| Comprehensive Utilization of Waste Resources Industry | 2 | 0.06 |
| Non-metallic Mineral Mining and Dressing Industry | 1 | 0.03 |
| Tobacco Products Industry | 1 | 0.03 |
| Total | 3513 | 100.00 |

**Table S3 The top five industries with ONID in manufacturing industry, 2006-2022 (n, %)**

| **Year** | **Top Industry** | **2nd Industry** | **3rd Industry** | **4th Industry** | **5th Industry** |
| --- | --- | --- | --- | --- | --- |
| 2006 | Railway, Shipbuilding, Aerospace, and Other Transportation Equipment Manufacturing Industry  (4, 26.7%) | Cultural, Educational, Arts, Sports, and Entertainment Products Manufacturing Industry  (3, 20.0%) | Other Manufacturing Industry  (3, 20.0%) | Non-metallic Mineral Products Industry  (2, 13.3%) | General Equipment Manufacturing Industry  (1, 6.7%) |
| 2007 | Railway, Shipbuilding, Aerospace, and Other Transportation Equipment Manufacturing Industry  (4, 33.3%) | Cultural, Educational, Arts, Sports, and Entertainment Products Manufacturing Industry  (3, 25.0%) | Metal Products Industry  (1, 8.3%) | Other Manufacturing Industry  (1, 8.3%) | Electrical Machinery and Equipment Manufacturing Industry  (1, 8.3%) |
| 2008 | Other Manufacturing Industry  (9, 25.7%) | Railway, Shipbuilding, Aerospace, and Other Transportation Equipment Manufacturing Industry  (6, 17.1%) | Cultural, Educational, Arts, Sports, and Entertainment Products Manufacturing Industry  (5, 14.3%) | Metal Products Industry  (4, 11.4%) | Leather, Fur, Feather and Related Products, and Footwear Industry  (2, 5.7%) |
| 2009 | Other Manufacturing Industry  (9, 23.1%) | Metal Products Industry  (6, 15.4%) | Cultural, Educational, Arts, Sports, and Entertainment Products Manufacturing Industry  (5, 12.8%) | Railway, Shipbuilding, Aerospace, and Other Transportation Equipment Manufacturing Industry  (3, 7.7%) | General Equipment Manufacturing Industry  (3, 7.7%) |
| 2010 | Other Manufacturing Industry  (9, 37.5%) | Leather, Fur, Feather and Related Products, and Footwear Industry  (4, 16.7%) | Petroleum, Coal, and Other Fuel Processing Industry  (4, 16.7%) | General Equipment Manufacturing Industry  (2, 8.3%) | Computer, Communication, and Other Electronic Equipment Manufacturing Industry  (1, 4.2%) |
| 2011 | Other Manufacturing Industry (20, 31.7%) | Metal Products Industry  (8, 12.7%) | Railway, Shipbuilding, Aerospace, and Other Transportation Equipment Manufacturing Industry  (8, 12.7%) | Computer, Communication, and Other Electronic Equipment Manufacturing Industry  (4, 6.3%) | Printing and Reproduction of Recorded Media Industry  (4, 6.3%) |
| 2012 | Metal Products Industry (21, 19.4%) | Other Manufacturing Industry  (14, 13.0%) | Railway, Shipbuilding, Aerospace, and Other Transportation Equipment Manufacturing Industry  (12, 11.1%) | Computer, Communication, and Other Electronic Equipment Manufacturing Industry  (12, 11.1%) | General Equipment Manufacturing Industry  (9, 8.3%) |
| 2013 | Other Manufacturing Industry (24, 20.9%) | Metal Products Industry  (17, 14.8%) | Railway, Shipbuilding, Aerospace, and Other Transportation Equipment Manufacturing Industry  (15, 13.0%) | Computer, Communication, and Other Electronic Equipment Manufacturing Industry  (14, 12.2%) | General Equipment Manufacturing Industry  (10, 8.7%) |
| 2014 | Computer, Communication, and Other Electronic Equipment Manufacturing Industry  (30, 16.9%) | Metal Products Industry  (24, 13.5%) | Electrical Machinery and Equipment Manufacturing Industry  (16, 9.0%) | General Equipment Manufacturing Industry  (13, 7.3%) | Railway, Shipbuilding, Aerospace, and Other Transportation Equipment Manufacturing Industry  (9, 5.1%) |
| 2015 | Computer, Communication, and Other Electronic Equipment Manufacturing Industry  (36, 16.2%) | Metal Products Industry  (27, 12.2%) | General Equipment Manufacturing Industry  (19, 8.6%) | Special Equipment Manufacturing Industry  (17, 7.7%) | Electrical Machinery and Equipment Manufacturing Industry  (14, 6.3%) |
| 2016 | Metal Products Industry  (55, 17.2%) | Computer, Communication, and Other Electronic Equipment Manufacturing Industry  (32, 10.0%) | Non-metallic Mineral Products Industry  (25, 7.8%) | Special Equipment Manufacturing Industry  (21, 6.6%) | Electrical Machinery and Equipment Manufacturing Industry  (20, 6.3%) |
| 2017 | Metal Products Industry  (57, 15.4%) | Computer, Communication, and Other Electronic Equipment Manufacturing Industry  (44, 11.9%) | Special Equipment Manufacturing Industry  (31, 8.4%) | General Equipment Manufacturing Industry  (30, 8.1%) | Electrical Machinery and Equipment Manufacturing Industry  (28, 7.6%) |
| 2018 | Metal Products Industry  (69, 16.4%) | Computer, Communication, and Other Electronic Equipment Manufacturing Industry  (50, 11.9%) | General Equipment Manufacturing Industry  (39, 9.3%) | Special Equipment Manufacturing Industry  (35, 8.3%) | Electrical Machinery and Equipment Manufacturing Industry  (34, 8.1%) |
| 2019 | Metal Products Industry  (76, 16.1%) | Computer, Communication, and Other Electronic Equipment Manufacturing Industry  (59, 12.5%) | General Equipment Manufacturing Industry  (46, 9.7%) | Special Equipment Manufacturing Industry  (45, 9.5%) | Electrical Machinery and Equipment Manufacturing Industry  (39, 8.2%) |
| 2020 | Metal Products Industry  (81, 16.8%) | Computer, Communication, and Other Electronic Equipment Manufacturing Industry  (65, 13.5%) | General Equipment Manufacturing Industry  (48, 10.0%) | Special Equipment Manufacturing Industry  (46, 9.5%) | Electrical Machinery and Equipment Manufacturing Industry  (41, 8.5%) |
| 2021 | Metal Products Industry  (89, 16.9%) | Computer, Communication, and Other Electronic Equipment Manufacturing Industry  (71, 13.5%) | General Equipment Manufacturing Industry  (55, 10.4%) | Special Equipment Manufacturing Industry  (50, 9.5%) | Electrical Machinery and Equipment Manufacturing Industry  (45, 8.5%) |
| 2022 | Metal Products Industry  (97, 17.1%) | Computer, Communication, and Other Electronic Equipment Manufacturing Industry  (77, 13.6%) | General Equipment Manufacturing Industry  (62, 10.9%) | Special Equipment Manufacturing Industry  (54, 9.5%) | Electrical Machinery and Equipment Manufacturing Industry  (49, 8.6%) |
